# Antimicrobial Activity and Phytochemical Analysis of Organic Extracts from *Cleome spinosa* Jaqc.

**DOI:** 10.3389/fmicb.2016.00963

**Published:** 2016-06-28

**Authors:** Ana P. Sant'Anna da Silva, Luís C. Nascimento da Silva, Caíque S. Martins da Fonseca, Janete M. de Araújo, Maria T. dos Santos Correia, Marilene da Silva Cavalcanti, Vera L. de Menezes Lima

**Affiliations:** ^1^Departamento de Bioquímica, Centro de Ciências Biológicas, Universidade Federal de PernambucoRecife, Brazil; ^2^Mestrado em Biologia Parasitária, Universidade CeumaSão Luís, Brazil; ^3^Departamento de Antibióticos, Centro de Ciências Biológicas, Universidade Federal de PernambucoRecife, Brazil; ^4^Departamento de Bioquímica, Centro de Ciências Biológicas, Universidade Federal de PernambucoRecife, Pernambuco, Brazil; ^5^Departamento de Micologia, Centro de Ciências Biológicas, Universidade Federal de PernambucoRecife, Pernambuco, Brazil

**Keywords:** plant-derived products, drug discovery, antibacterial agents, synergistic action, *S. aureus*

## Abstract

Due to the use of *Cleome spinosa* Jacq. (Cleomaceae) in traditional medicine against inflammatory and infectious processes, this study evaluated the *in vitro* antimicrobial potential and phytochemical composition of extracts from its roots and leaves. From leaves (L) and roots (R) of *C. spinosa* different extracts were obtained (cyclohexane: ChL and ChR; chloroform: CL and CR; ethyl acetate: EAL and EAR, methanol: ML and MR). The antimicrobial activity was evaluated by the broth microdilution method to obtain the minimum inhibitory (MIC) and microbicidal (MMC) concentrations against 17 species, including bacteria and yeasts. Additionally, antimicrobial and combinatory effects with oxacillin were assessed against eight clinical isolates of *Staphylococcus aureus*. All *C. spinosa* extracts showed a broad spectrum of antimicrobial activity, as they have inhibited all tested bacteria and yeasts. This activity seems to be related to the phytochemicals (flavonoid, terpenoids and saponins) detected into the extracts of *C. spinosa*. ChL and CL extracts were the most actives, with MIC less than 1 mg/mL against *S. aureus, Bacillus subtilis*, and *Micrococcus luteus*. It is important to note that these concentrations are much lower than their 50% hemolysis concentration (HC_50_) values. Strong correlations were found between the average MIC against *S. aureus* and their phenolic (*r* = −0.89) and flavonoid content (*r* = −0.87), reinforcing the possible role of these metabolite classes on the antimicrobial activity of *C. spinosa* derived extracts. Moreover, CL and CR showed the best inhibitory activity against *S. aureus* clinical isolates, they also showed synergistic action with oxacillin against all these strains (at least at one combined proportion). These results encourage the identification of active substances which could be used as lead(s) molecules in the development of new antimicrobial drugs.

## Introduction

Microbial resistance to antibiotics is one of the most serious public health problems, especially in developing countries where infectious diseases still represent a major cause of human mortality (World Health Organization, 2014). Among the microorganisms that represent a significant health threat, *Staphylococcus aureus* is highlighted as this species is responsible for a number of human illness conditions, such as skin infections and septicemia (Adhikari et al., 2012). Among fungal pathogens, the genus *Candida* also has high clinical relevance and it is responsible for a wide variety of infections, from superficial mucocutaneous to more invasive diseases (Kim and Sudbery, 2011). Approximately 75% of women, at least once in their life, develop candidiasis caused by *C. albicans, C. glabrata, C. parapsilosis, C. tropicalis*, and/or *C. krusei* (Simonetti et al., 2014). This issue encourages the search for novel antimicrobial agents.

One of the oldest forms of medical practice is the use of plants for therapeutic purposes; teas, syrups, tinctures, among others have been used as medicines and in many cases come to be the sole therapeutic resource of certain communities and ethnic groups (Amorozo, 2002; Oliveira et al., 2012). Thus, knowledge about the therapeutic potential of plants is of great scientific and medical interest, as an effective alternative to the battle against resistant microorganisms (dos Santos et al., 2015).

Some species of the genus *Cleome* (Cleomaceae) have been investigated for medical properties and some of them had their anti-inflammatory (Albarello et al., 2013), analgesic (Bose et al., 2007), and antimicrobial (Sudhakar et al., 2006; McNeil et al., 2010) activities evaluated. *C. spinosa* Jacq is a perennial herb that grows in the Central-West, Northeast, North and Southeast of Brazil and is known in Brazil as “Mussambê.” Its leaves and flowers have been used in traditional medicine: leaves infusion is used in the treatment of asthma, cough, and bronchitis, while flowers infusion is used against fever (Agra et al., 2007). So far, some pharmacological actions have been proven such as antimicrobial, antinociceptive, anti-inflammatory, anthelmintic (McNeil et al., 2010; Albarello et al., 2013; Andrade et al., 2014). Regarding the antimicrobial activity, it is only reported for essential oils from leaves, which significantly inhibit *Streptococcus pyogenes* Group A (McNeil et al., 2010). Based on the uses of *C. spinosa* in folk medicine, it is attractive to analyze the antimicrobial potential of other tissue, using also different extraction methods. In this sense, the first step of this study was to evaluate the antimicrobial activity of different extracts from leaves and roots of *C. spinosa* against a set of 17 microbial species. The phytochemical constituents of each extract were determined and correlated with the antimicrobial action. The active extracts were tested against clinical isolates of *S. aureus* and their combinatory effects with oxacillin were also evaluated.

## Materials and methods

### Plant material

The leaves and roots of *C. spinosa* were collected at *Universidade Federal Rural de Pernambuco* – *Dois Irmãos* (Latitude 8° 01′22.3″; Longitude: 34° 57′15.8″). The botanical material was identified by Dr. Marlene Carvalho de Alencar Barbosa and deposited in the Herbarium UFPE - Geraldo Mariz, at the Department of Botany, *Universidade Federal de Pernambuco* (UFPE), under the voucher number 76,556.

### Extract preparation

Samples (100 g) from each tissue of *C. spinosa* were dried and milled and separately subjected to Soxhlet extraction using an eluotropic series of solvents (in the following order: cyclohexane, chloroform, ethyl acetate and methanol) at a temperature below of the boiling point of each solvent. All samples were subjected to saturation at reflux for 24 h. After this time, the extracts were filtered through a Whatman filter paper No 1. All extracts from leaves (L) or roots (R) (cyclohexane: ChL and ChR; chloroform: CL and CR; ethyl acetate: EAL and EAR: methanol: ML and MR) were concentrated until the complete removal of the solvent on a rotating evaporator at 45°C (under reduced pressure) and stored for later analyses. For phytochemical analysis the extracts were dissolved in ethanol (1 mg/mL), while they were solubilized in a sterile dimethylsulfoxide solution (DMSO; 10% in water) at the concentration of 100 mg/mL for all biological assays.

### Phytochemical analysis

An aliquot (10 μL) of each extract (1 mg/mL in ethanol) obtained from leaves and roots of *C. spinosa* was subjected to qualitative phytochemical analysis to ascertain the presence of secondary metabolites such as: alkaloids, coumarins, anthracene and cinnamic acid derivatives, flavonoids, monoterpenoides, sesquiterpenoids, diterpenoids (Wagner and Bladt, 1996); tannins, triterpenoids and steroids (Harborne, 1998); proanthocyanidins and leucoanthocyanidins (Robertson et al., 1957); reducing sugars (Russell and Morris, 1982) and saponins (Costa, 2002) respectively. The compounds classes were visualized as aid thin layer chromatography (TLC) on silica gel 60 F254 (Merck, Germany), different systems of development and adequate visualization techniques were used: Dragendorff, NEU-PEG, KOH-Ethanol, Liebermann-Burchard, vanillin-sulfuric acid and others reagents, according to the respective method.

#### Determination of total phenolic content

The total phenol content in each extract was determined as reported by Li et al. (2008). Samples (200 μL) of each extract at 1 mg/mL were mixed with 1 mL of diluted Folin-Ciocalteu reagent (1:10 in water). After 4 min, this mixture received 800 μL of saturated solution sodium carbonate (75 mg/mL), followed by 2 h of incubation at room temperature (protected from light). The absorbance was measured at 765 nm. Gallic acid (0–500 mg/L) was used as standard in a calibration curve. The results were expressed as mg of gallic acid equivalents (GAE)/g dry weight of plant extract.

#### Determination of flavonoids

The quantification of total flavonoid content in each extract followed the methodology proposed by Woisky and Salatino (1998). In briefly, to 0.5 mL of diluted samples (1 mg) was added 0.5 mL of 2% AlCl_3_ (w/v) solution prepared in methanol. After 30 min of incubation at room temperature, protected from light, the absorbance was measured at 420 nm. All measurements were done in triplicate. The results were expressed as mg of quercetin equivalent (QE)/g dry weight of plant extract.

### Antimicrobial assays

#### Microorganisms

Twenty bacterial strains from 12 species (*Bacillus subtilis, Enterococcus faecalis, Micrococcus luteus, Mycobacterium smegmatis, Staphylococcus epidermidis, Streptococcus mutans, Escherichia coli, Klebsiella pneumoniae, Proteus mirabilis, Pseudomonas aeruginosa, Salmonella enteritidis*) provided by the Culture Collection UFPEDA (Department of Antibiotics, UFPE) and five *Candida* species (*Candida albicans, Candida krusei, Candida glabrata, Candida parapsilosis, Candida tropicalis*) obtained from the Culture Collection URM (Department of Mycology, UFPE) were used for the antimicrobial tests, according to Table 1.

**Table 1:** **Microorganisms used in this study**.

**Bacteria**	**UFPEDA No**.	**Bacteria**	**UFPEDA No**.	**Yeasts**	**URM No**.
*Bacillus subtilis*	086	*Staphylococcus aureus*	002	*Candida albicans*	5852
*Enterococcus faecalis*	138	*Staphylococcus aureus*	670^1^	*Candida krusei*	6391
*Micrococcus luteus*	100	*Staphylococcus aureus*	683^1^	*Candida glabrata*	6343
*Mycobacterium smegmatis*	071	*Staphylococcus aureus*	689^1^	*Candida parapsilosis*	6557
*Staphylococcus epidermidis*	183	*Staphylococcus aureus*	700^1^	*Candida tropicalis*	6711
*Streptococcus mutans*	766	*Staphylococcus aureus*	705^1^		
*Escherichia coli*	224	*Staphylococcus aureus*	709^1^		
*Klebsiella pneumoniae*	396	*Staphylococcus aureus*	726^1^		
*Proteus mirabilis*	737	*Staphylococcus aureus*	731^1^		
*Pseudomonas aeruginosa*	416				
*Salmonella enteritidis*	414				

1 *Source of S. aureus strains; 670, Urine sample; 683, Bone fragment; 689, Ulcer secretion; 700, Ulcer secretion; 705, Surgical wound; 709, Purulent exudates; 726, Nasal secretion; 731, Surgical wound secretion*.

#### Determination of minimum inhibitory concentration (MIC) and minimal microbicidal concentration (MMC)

The minimum inhibitory (MIC) and minimal microbicidal (MMC) concentrations were determined by the broth microdilution method (Oliveira et al., 2012). Sabouraud broth was used for yeasts and Mueller Hinton broth for bacterial strains, except for *Streptococcus mutans*, which was cultivated in Brain-Heart infusion broth (BHI). Tested microorganisms were standardized by the Mcfarland turbidity scale equivalent to the tube 0.5, corresponding to a concentration of approximately 10^7^ CFU/mL for yeasts and 10^8^ CFU/mL for bacteria. Serial dilutions of all extracts were made in 96-well plates to obtain concentrations ranging from 50 to 0.09 mg/mL. Following that, each well received 10 μL of microorganism suspensions and the plates were incubated at 25°C for 48 h for yeasts and at 37°C for 24 h for bacteria. After each period, 15 μL of 0.01% resazurin was added, as a colorimetric indicator of cell viability. Then, the microplates were re-incubated for 4 h and the lowest concentration of extract that inhibited microbial growth was recorded as the MIC. Then 50 μL of the solution from each inhibited well was collected and transferred to agar plates and re-incubated as described above for the yeasts and bacteria. The complete absence of growth on the agar surface with the lowest concentration of the sample was defined as the MMC. Commercially available antibiotics were used as positive control for bacteria (ampicillin and oxacillin) and yeast (amphotericin B, fluconazole and itraconazole). Sterile DMSO aqueous solution (10%) was used as negative control.

#### Evaluation of combinatory effects of chloroform extracts and oxacillin

Combinatory effects between chloroform extracts of *C. spinosa* and oxacillin were assessed using different *S. aureus* strains (UFPEDA 670, 672, 683, 700, 705, 709, 726, and 731). Briefly, samples were combined at different proportions of plant extract and drug (1:1, 1:2 and 1:3; final volume: 200 μL) using stock solutions of each extract (6.25 mg/mL) and oxacillin (0.5 mg/mL). Serial dilutions of each combination were made in 96-well plates to obtain concentrations ranging from 3.15 mg/mL to 0.003 mg/mL and 0.25 mg/mL to 0.0002 mg/mL; 2.08 mg/mL to 0.002 mg/mL and 0.33 mg/mL to 0.0003 mg/mL; and 1.56 mg/mL to 0.001 mg/mL and 0.375 mg/mL to 0.0004 mg/mL for plant extract and drug combined at 1:1, 1:2 and 1:3. The antibacterial activity was performed as described for MIC determination. The Fractional Inhibitory Concentration (ΣFIC) was calculated according to the equation:

ΣFIC = (MICE + D/MICE) + (MICD + E/MICD) MICE + D: minimal inhibitory concentration of extract in combination with oxacillin; MICD+E: minimal inhibitory concentration of oxacillin in combination with extract. Results were considered: synergistic (ΣFIC < 0.5); additive (0.5 < ΣFIC < 1); non-interactive (1 < ΣFIC < 4); or antagonist (ΣFIC > 4) (Odds, 2003; Vuuren and Viljoen, 2011).

### Hemolytic assay

Blood (5–10 mL) was obtained from healthy nonsmoking volunteers by venipuncture, after a written informed consent was obtained. Human erythrocytes from citrated blood were immediately isolated by centrifugation at 1500 rpm for 10 min at 4°C. After removal of plasma and buffy coat, the erythrocytes were washed three times with phosphate-buffered saline (PBS; pH 7.4) and then resuspended using the same buffer and a 1% erythrocyte suspension was prepared. The hemolytic activity of the crude extract was tested under *in vitro* conditions. Each tube received 1.1 mL of erythrocyte suspension and 0.4 mL of extract of various concentrations (31.25–1000 μg/mL) were added. The negative control was only solvent and the positive control received 0.4 mL of Triton X-100. After 60-min incubation at room temperature, cells were centrifuged and the supernatant was used to measure the absorbance of the liberated hemoglobin at 540 nm (Oliveira et al., 2012). The average value was calculated from triplicate assays. The relative hemolytic activity was expressed in relation to Triton X-100 and calculated by the following formula:

Relative hemolytic activity (%)=[(As−Ab).100]/(Ac−Ab)

Where Ab was the absorbance of the control (blank, without extract), As was the absorbance in the presence of the extract, and Ac was the absorbance in the presence of Triton X-100. Hemolysis concentration was calculated.

### Statistical analyses

Each experiment was performed in triplicate in at least two independent experiments. Statistical analyses were performed by One-way analysis of variance (ANOVA). All analyses were carried out using GraphPrism, version 4. Differences were considered significant at *p* < 0.05. The concentration needed for 50% of hemolysis (HC_50_ values) was calculated graphically by linear regression analysis. The correlation indices were calculated using the Pearson coefficient (*r*) and were classified as strong (*r*: −1.0 to −0.7), moderate (*r*: −0.69 to −0.5), week (*r*: −0.49 to −0.3) or negligible (*r* < −0.3).

## Results

### Phytochemical analyses of the samples

Analysis of the yield of all the extractions showed that the *C. spinosa* leaves had a better result, in comparison with the roots. With regards to the solvents, methanol extracts showed the better yield, when compared to the hexane, chloroform and ethyl acetate extracts, as showed in Table 2.

**Table 2:** **Phytochemical analyses of organic extracts from leaves and roots of *C. spinosa***.

**Extract**	**Yield (%)**	**Quantitative analyses**		**Phytochemical screen**	
		**Total phenolic content (mg GAE/g)**	**Flavonoid content (mg QE/g)**	**Positive tests for**	**Negative test for**
ChL	3.77	131.3 ± 0.7	11.9 ± 0.1	Reducing sugars, anthracene derivatives, flavonoids, tannins, monoterpenes, sesquiterpenes, diterpenes, triterpenes, steroids, proanthocyanidins and leucoanthocyanidins	Alkaloids, coumarins, cinnamic acid derivatives, saponins,
CL	2.15	201.7 ± 0.8	153.7 ± 0.6	Reducing sugars, anthracene derivatives, flavonoids, tannins, monoterpenes, sesquiterpenes, diterpenes, triterpenes, steroids, proanthocyanidins and leucoanthocyanidins	Alkaloids, coumarins, cinnamic acid derivatives, saponins,
EAL	2.81	173.6 ± 0.5	48.4 ± 0.2	Reducing sugars, anthracene derivatives, flavonoids, tannins, triterpenes, steroids, proanthocyanidins and leucoanthocyanidins	Alkaloids, coumarins, cinnamic acid derivatives, saponins, monoterpenes, sesquiterpenes, diterpenes
ML	6.23	255.7 ± 0.3	71.8 ± 0.8	Reducing sugars, anthracene derivatives, flavonoids, tannins, cinnamic acid derivatives, saponins, monoterpenes, sesquiterpenes, diterpenes,	Alkaloids, coumarins, triterpenes, steroids, proanthocyanidins and leucoanthocyanidins
ChR	0.38	72.73 ± 1.43	4.7 ± 0.1	Reducing sugars, anthracene derivatives, flavonoids, tannins, coumarins, monoterpenes, sesquiterpenes, diterpenes, triterpenes, steroids	Alkaloids, cinnamic acid derivatives, saponins, proanthocyanidins and leucoanthocyanidins
CR	0.27	215.2 ± 0.6	24 ± 0.0	Reducing Sugars, anthracene derivatives, flavonoids, tannins, monoterpenes, sesquiterpenes, diterpenes, triterpenes, steroids	Alkaloids, coumarins, cinnamic acid derivatives, saponins, proanthocyanidins and leucoanthocyanidins
EAR	0.1	256.94 ± 0.5	36.0 ± 0.2	Reducing sugars, anthracene derivatives, flavonoids, tannins, saponins, monoterpenes, sesquiterpenes, diterpenes, triterpenes, steroids	Alkaloids, coumarins, cinnamic acid derivatives, proanthocyanidins and leucoanthocyanidins
MR	3.36	125.9 ± 0.70	7.9 ± 0.1	Reducing sugars, anthracene derivatives, flavonoids, tannins, coumarins, cinnamic acid derivatives, saponins, monoterpenes, sesquiterpenes, diterpenes,	Alkaloids, triterpenes, steroids, proanthocyanidins and leucoanthocyanidins

*Cyclohexane extracts, ChL and ChR; chloroform extracts, CL and CR; ethyl acetate extracts, EAL and EAR: methanolic extracts, ML and MR*.

The qualitative phytochemical analysis detected the presence of reducing sugars, antracenic derivatives, flavonoids and terpenes into all extracts. Additionally, ChL, CL, CR and EAR also presented monoterpenes, sesquiterpenes, diterpenes, triterpenes, and steroids; the EAL showed saponins, triterpenes, and steroids; ML exhibited saponins, monoterpenes, and sesquiterpenes; ChR revealed coumarins, triterpenes, and steroids; and MR presented coumarins, cinnamic acid derivatives, saponins, monoterpenes, sesquiterpenes, and diterpenes.

The estimation of total phenolic content revealed that EAR (256.94 ± 0.48 mg GAE/g), ML (255.69 ± 0.28 mg GAE/g) and CR (215.23 ± 0.64 mg GAE/g) exhibited the highest phenolic content (*p* < 0.05). The other extracts showed phenolic content values ranging from 72.73 mg GAE/g to 201.71 mg GAE/g (Table 2). On the hand, CL and ML showed the highest flavonoids content with values of 153.70 ± 0.58 mg QE/g and 71.83 ± 0.76 mg QE/g, respectively. The total phenolic and flavonoids contents showed a weak correlation (*r* = 0.48).

### Antimicrobial screening

The antimicrobial activity of the organic extracts from leaves and roots of *C. spinosa* are presented in Tables 3–**5**. Overall, all extracts from both leaves and roots of *C. spinosa* exhibited antimicrobial activity with broad spectrum, as they inhibited all tested bacteria and yeasts.

**Table 3:** **Antimicrobial Activity of organic extracts from leaves and roots of *C. spinosa* against selected Gram-positive bacteria**.

***C. spinosa* Extracts**	***Bacillus subtilis***		***Enterococcus faecalis***		***Micrococcus luteus***		***Mycobacterium smegmatis***		***Staphylococcus aureus***		***Staphylococcus epidermidis***		***Streptococcus mutans***	
	**MIC**	**MMC**	**MIC**	**MMC**	**MIC**	**MMC**	**MIC**	**MMC**	**MIC**	**MMC**	**MIC**	**MMC**	**MIC**	**MMC**
ChL	0.19	3.12	25	25	0.78	1.56	6.25	12.5	0.09	0.19	6.25	12.5	6.25	25
CL	1.56	12.5	25	25	1.56	3.12	12.5	12.5	0.78	1.56	6.25	6.25	12.5	50
EAL	1.56	1.56	12.5	12.5	0.78	3.12	6.25	12.5	6.25	50	3.12	6.25	12.5	25
ML	6.25	6.25	25	25	1.56	3.12	12.5	25	6.25	>50	6.25	6.25	6.25	>50
ChR	0.09	0.19	25	25	0.39	0.78	12.5	25	3.12	12.5	6.25	12.5	12.5	50
CR	0.09	0.19	25	25	0.39	0.78	6.25	12.5	0.78	3.12	12.5	12.5	12.5	50
EAR	0.78	0.78	6.25	6.25	0.39	1.56	6.25	12.5	1.56	12.5	3.12	6.25	6.25	25
MR	3.12	3.12	25	25	0.78	1.56	12.5	25	6.25	25	6.25	6.25	12.5	>50
Control	MIC		MIC		MIC		MIC		MIC		MIC		MIC	
AMP	0.002		0.001		0.00012		0.004		0.004		0.004		0.00025	
OXA	0.025		0.001		0.00012		0.002		0.00012		0.0006		0.00012	

*MIC, Minimal Inhibitory Concentration; MMC, Minimal Microbicidal Concentration; MIC and MMC values are expressed in mg/mL. Cyclohexane extract, ChL; chloroform extract, CL; ethyl acetate extract, EAL: methanolic extract, ML. AMP, ampicillin; OXA, oxacillin*.

Furthermore, the best antibacterial results observed were provided by the extracts obtained using ciclohexane and chloroform, whose MIC ranged from 0.09 mg/mL to 12.5 mg/mL (Tables 3, 4). We considered MIC less than or equal to 1.56 mg/mL as considerable inhibition. Among extracts from leaves, ChL presented the best antibacterial activity against *S. aureus, B. subtilis* and *M. luteus* with MIC values of 0.09 mg/mL, 0.19 mg/mL and 0.78 mg/mL, respectively. CL was also more active against these same microorganisms (MIC ≤ 1.56 mg/mL). EAL best inhibited *M. luteus* (MIC = 0.78 mg/mL) and *B. subtilis* (MIC = 1.56 mg/mL) and ML best inhibited *M. luteus* (MIC = 1.56 mg/mL). In the case of the root extracts, ChR and CR were particularly effective against *B. subtilis* (MIC of 0.09 mg/mL for both extracts) and *M. luteus* (MIC of 0.39 mg/mL for both extracts). Additionally, CR demonstrated anti-*S. aureus* potential (MIC = 0.78 mg/mL). EAR showed markedly activity against *M. luteus* (MIC = 0.39 mg/mL), *B. subtilis* (MIC = 0.78 mg/mL), *S. aureus* (MIC = 1.56 mg/mL). The MR extract exhibited its best activity against *M. luteus* (MIC = 0.78 mg/mL).

**Table 4:** **Antimicrobial Activity of organic extracts from leaves and roots of *C. spinosa* against selected Gram-negative bacteria**.

***C. spinosa* Extracts**	***Escherichia coli***		***Klebsiella pneumoniae***		***Proteus mirabilis***		***Pseudomonas aeruginosa***		***Salmonella enteritidis***	
	**MIC**	**MMC**	**MIC**	**MMC**	**MIC**	**MMC**	**MIC**	**MMC**	**MIC**	**MMC**
ChL	12.5	25	12.5	>50	12.5	25	6.25	6.25	6.25	25
CL	25	25	25	>50	12.5	25	3.12	6.25	12.5	25
EAL	6.25	12.5	12.5	>50	6.25	12.5	3.12	12.5	12.5	50
ML	12.5	50	12.5	>50	12.5	50	3.12	3.12	12.5	>50
ChR	12.5	50	25	50	12.5	25	6.25	12.5	12.5	50
CR	12.5	50	12.5	50	6.25	25	6.25	50	12.5	25
EAR	12.5	50	12.5	12.5	12.5	12.5	12.5	50	6.25	12.5
MR	12.5	25	12.5	50	25	25	12.5	50	12.5	50
Control	MIC		MIC		MIC		MIC		MIC	
AMP	0.004		0.008		0.004		0.002		>0.128	
OXA	0.008		0.008		0.00025		0.004		0.064	

*MIC, Minimal Inhibitory Concentration. MMC, Minimal Microbicidal Concentration. MIC and MMC values are expressed in mg/mL. Cyclohexane extract, ChR; chloroform extract, CR; ethyl acetate extract, EAR; methanolic extract, MR*.

The antifungal activity of the *C. spinosa* extracts is presented in Table 5. The leaf extracts showed MIC values ranging from 3.12 to 12.5 mg/mL and MMC between 6.25 and 50 mg/mL. The extracts from leaves were less active, as they inhibited yeasts in concentrations between 6.25 and 12.5 mg/mL and killed them at dosage from 12.5 mg/mL. Among leaves extracts, the CL was the most active against all *Candida* species tested, except *C. krusei*, followed by ChL. The extracts from the root part showed a circumspect activity, with ChR effective against *C. glabrata, C. krusei* and *C. parasiplosis*; CR against *C. krusei* and *C. parasiplosis*; EAR against *C. parasiplosis*; and MR against *C. tropicalis* and *C. parasiplosis* (Table 5).

**Table 5:** **Antimicrobial Activity of organic extracts from leaves and roots of *C. spinosa* against *Candida* spp**.

***C. spinosa***	***Candida albicans***		***Candida glabrata***		***Candida krusei***		***Candida parapsilosis***		***Candida tropicalis***	
**Extracts**	**MIC**	**MMC**	**MIC**	**MMC**	**MIC**	**MMC**	**MIC**	**MMC**	**MIC**	**MMC**
ChL	12.5	12.5	3.12	25	12.5	25	6.25	25	6.25	50
CL	3.12	12.5	3.12	50	12.5	25	3.12	12.5	3.12	50
EAL	6.25	12.5	3.12	25	12.5	25	6.25	12.5	6.25	50
ML	6.25	12.5	6.25	25	12.5	25	6.25	6.25	3.12	50
ChR	12.5	25	6.25	25	6.25	25	6.25	25	12.5	12.5
CR	12.5	50	12.5	>50	6.25	25	6.25	25	12.5	50
EAR	12.5	12.5	12.5	25	12.5	25	6.25	12.5	12.5	25
MR	12.5	12.5	12.5	50	6.25	25	6.25	25	6.25	25
Control	MIC		MIC		MIC		MIC		MIC	
FLU	0.0005		0.001		0.002		0.002		0.001	
ITCZ	0.00003		0.00012		0.00006		0.00006		0.00006	
AMP-B	0.002		0.00025		0.00012		0.001		0.001	

*MIC, Minimal Inhibitory Concentration; MMC, Minimal Microbicidal Concentration; MIC and MMC values are expressed in mg/mL. Cyclohexane extracts, ChL and ChR; chloroform extracts, CL and CR; ethyl acetate extracts, EAL and EAR; methanolic extracts, ML and MR*.

*FLU, fluconazole; ITCZ, itraconazole; AMP-B, amphotericin B*.

### Hemolytic assay

**Figure 1:**
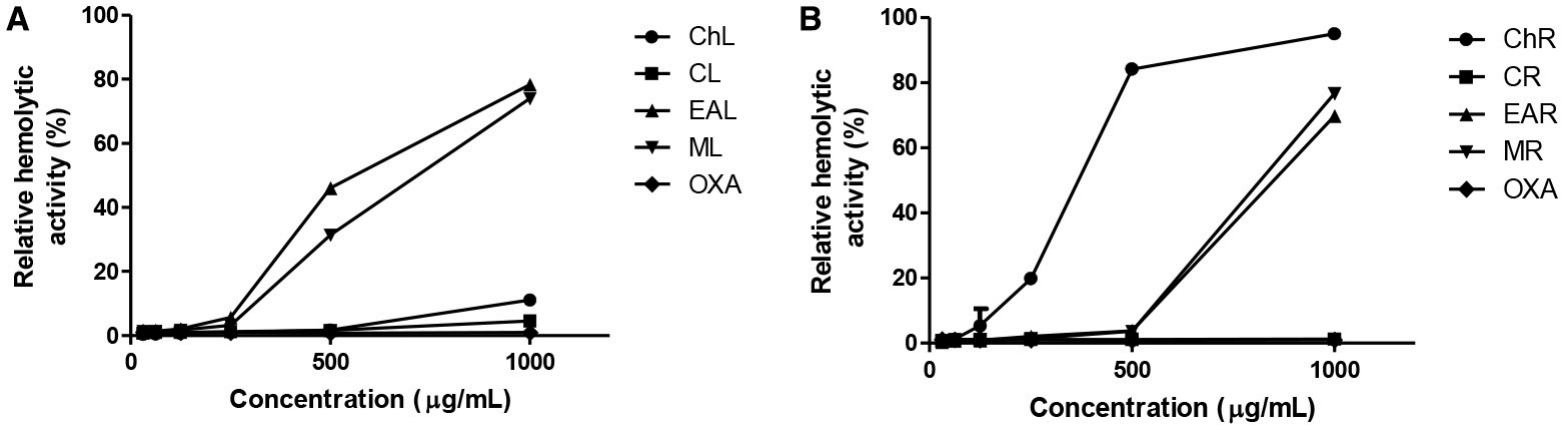
**Hemolytic Activity of organic extracts from leaves (A) and roots (B) of *C. spinosa***. Cyclohexane extracts, ChL and ChR; chloroform extracts, CL and CR; ethyl acetate extracts, EAL and EAR; methanolic extracts, ML and MR; Oxacillin, OXA. The hemotytic activity in relation to Triton X-100 (0.4–1.1 mL of erythrocyte suspension).

The hemolytic activity of each extract was performed using fresh human erythrocytes and they showed low cytotoxicity (Figure 1). Even at the highest tested concentration (1000 μg/mL), the extracts CR, CL and ChL induced low levels of hemolysis (1.18, 4.54, and 11.13%, respectively). Regarding the HC_50_, ChR was the most toxic extract (470.88 μg/mL), followed by EAL (648.97 μg/mL), ML (725.58 μg/mL), EAR (870.72 μg/mL), MR (808.51 μg/mL). The less toxic extracts had theoretical HC_50_ values of 4773.602 μg/mL, 13409.35 μg/mL and 151.389.2 μg/mL for ChL, CL and MR, respectively. In addition, these HC_50_ values were not correlated with either flavonoid (*r* = 0.23) or phenolic content (*r* = −0.10).

### Anti-*S. aureus* activity and combinatory effects with oxacillin

**Figure 2:**
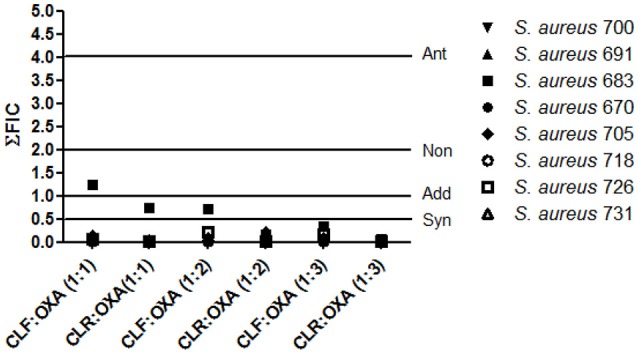
**Combinatory effect of Oxacillin and chloroform extracts from leaves (CLF) and roots (CLR) of *C. spinosa* against clinical isolates of *Staphylococcus aureus***. Each extract solution (6.25 mg/mL) was mixed with oxacillin solution (OXA; 0.5 mg/mL) at different proportion extract/drug (1:1, 1:2; 1:3). The Fractional Inhibitory Concentration (ΣFIC) was calculated as demonstrated at methods section. Interpretation: Syn, synergistic effect (ΣFIC < 0.5); Add, additive effect (0.5 < ΣFIC < 1); Non, non-interactive effect (1 < ΣFIC < 4); Ant, antagonist effect (ΣFIC > 4)

Given the high medical importance of the antibiotic resistant in *S. aureus* strains, the action of the extracts was evaluated in association with oxacillin against clinical isolates (Table 6). The extracts were effective against all strains. However, those obtained using chloroform showed the best efficiencies (*p* < 0.05), with MIC_50_ values (concentration able to inhibit 50% of the strains) of 0.78 mg/mL for leaves and 3.12 mg/mL for roots; while the cyclohexane extracts exhibited MIC_50_ values of 6.25 mg/mL. Then, the combinatorial effect of the chloroform extracts with oxacillin was evaluated. Almost all combinations had synergistic activities, the exceptions were the non-interactive (for the combination CLF:OXA at 1:1) and additive effects (CLR:OXA at 1:1; and CLF:OXA at 1:2) observed against the isolate 683 (Figure 2). CLR only exhibited synergetic and additive effects.

**Table 6:** **Antimicrobial Activity of organic extracts from leaves and roots of *C. spinosa* against *Staphylococcus aureus***.

***S. aureus***	**Oxacillin**	***C. spinosa* Extracts**											
**(UFPEDA No.)**		**ChL**			**CL**			**ChR**			**CR**		
	**MIC**	**MIC**	**MMC**	**MMC/MIC**	**MIC**	**MMC**	**MMC/MIC**	**MIC**	**MMC**	**MMC/MIC**	**MIC**	**MMC**	**MMC/MIC**
670	0.0025	6.25	25	4	0.19	0.78	4	6.25	25	4	6.25	25	4
683	0.0050	1.56	3.12	2	0.39	1.56	4	12.5	50	4	6.25	12.5	2
691	0.0050	3.12	25	4	0.19	1.56	4	6.25	25	4	1.56	12.5	4
700	0.0050	6.25	50	4	3.12	25	4	6.25	25	4	6.25	6.25	1
705	0.0050	6.25	12.5	2	3.12	6.25	2	6.25	12.5	2	3.12	3.12	1
718	0.0025	6.25	25	4	0.78	6.25	4	12.5	50	4	3.12	6.25	2
726	0.0025	12.5	50	4	6.25	12.5	2	6.25	12.5	2	6.25	12.5	2
731	0.0050	12.5	50	4	6.25	12.5	2	6.25	25	4	3.12	6.25	2

*MIC, Minimal Inhibitory Concentration; MMC, Minimal Microbicidal Concentration; MIC and MMC values are expressed in mg/mL. Cyclohexane extracts, ChL and ChR; chloroform extracts, CL and CR*.

## Discussion

Considering that the World is facing a growing number of multidrug-resistant microorganisms, numerous studies have been conducted in order to select new compounds, such as those from natural resources which are of extremely importance (World Health Organization, 2014; dos Santos et al., 2015). The plants are, admittedly, a valuable reservoir of bioactive compounds of substantial medical importance (Harvey et al., 2015). So, this study has evaluated the antimicrobial activity of *C. spinosa*, a species known for their use in traditional medicine to treat infections. Some studies have shown antimicrobial, insecticidal, and antioxidant activities in essential oils obtained from their aerial parts of *C. spinosa* (McNeil et al., 2010), others had evaluated the anti-inflammatory and antinociceptive effects of methanol extracts from different ways of cultivation (Albarello et al., 2013).

Qualitative phytochemical analysis clearly demonstrated the presence of number important active constituents and revealed that both *C. spinosa* tissues have similar phytochemical constitution. Only the extracts from leaves (cyclohexane, chloroform, ethyl acetate) showed proanthocyanidins and leucoanthocyanidins. Indeed, all kind of metabolite classes detected into the samples (saponins, flavonoids, tannins, coumarins and terpenoids) are well known to have significant inhibitory action against bacteria and fungi (Hayek et al., 2013). This fact can justify why all extracts showed antimicrobial activity. On the other hand, quantitative assays revealed that the extracts have different phenolic and flavonoid contents which can be correlated with each biological activity. For example, for roots derived extracts a strong direct correlation was found between their average MIC values (for all tested bacteria) and phenolic (*r* = −0.87) and flavonoid contents (*r* = −0.96). For leaves extracts inverse strong and moderate correlations were found (*r*-value of 0.56 and 0.88 for correlation with phenolic and flavonoid contents, respectively), suggesting that the antimicrobial activity might be an effect of other types of compounds. It is important to note that when the correlations between polyphenols/flavonoid content were determined using and average MIC or HC_50_, a negative *r*-value (*r* = −1) is considered as perfect direct correlation.

On the basis of the results shown in Tables 4, 5, *C. spinosa* extracts demonstrated to be more active on Gram-positive than on Gram-negative bacteria. This was not a surprise since previous studies have reported that generally plant extracts are usually more active against Gram-positive bacteria than Gram-negative bacteria, and the susceptibility may be due to structural differences in the cell wall of these classes of bacteria. Cells of Gram-negative bacteria are surrounded by an additional outer membrane, which provide them with a hydrophilic surface that functions as a permeability barrier for many substances including natural compounds (Hemaiswarya et al., 2008; Briers and Lavigne, 2015). Additional contribution to intrinsic resistance in Gram-negative bacteria is provided by efflux pumps (Eps) which actively pump out a broad spectrum of compounds (such as antibiotics, toxins, β-lactamase inhibitors, dyes, detergents, lipids, and molecules involved in quorum sensing) from the periplasm to the outside of the cell. The overexpression of EPs (such as Resistance-Nodulation-Division-type efflux pumps) is recognized as a major component in the development of the multidrug resistance phenotype in Gram-negative bacteria (Opperman and Nguyen, 2015; Venter et al., 2015). The ineffectiveness of plant compounds toward Gram-negative pathogens has been proposed to be strongly related to EPs as the combination of plant antimicrobials with EPs inhibitors leads to a striking increase in antimicrobial activity (Tegos et al., 2002).

Although the of action mechanisms natural products are distinct, the cytoplasmic membrane ranks as the most common site of action for secondary metabolites. They usually act through cell lysis, triggering the leakage of cellular contents and consequently cell death (Da Silva et al., 2013). The interaction with genetic material and protein synthesis is also a possible factor regarded to the promotion of the therapeutic action. In this case, when there is a contact with the genetic material, the compound is able to promote changes in the genetic machinery, whose result is ineffective transcription and disturbance of vital functions for the cell (Hayek et al., 2013; Gyawali and Ibrahim, 2014). The phenolic compounds (polyphenols, tannins, and flavonoid) can act at two different levels: the cell membrane and cell wall of the microorganisms (Taguri et al., 2006). They can also penetrate into bacterial cells and coagulate cell content (Tian et al., 2009). The antimicrobial property of saponins is due to a lipophilic portion into its structure (aglycon or sapogenin) and a hydrophilic core comprising one or more sugars (Costa et al., 2010).

Among all extracts, ChL and CR were the most active and inhibited the growth of *B. subtilis, M. luteus, S. aureus*. They demonstrated an average MIC of 0.35 mg/mL and 0.42 mg/mL (these values were not significantly different at the *p* > 0.05 level), and a predominantly bactericidal behavior: MMC/MIC ratio < 4 (Pankey and Sabath, 2004). To those same pathogens, the average MIC for ChL was up to 13-fold lower than the other extracts, while for CR was up to 8-fold lower. These extracts, qualitatively, showed the same phytochemical composition than the others, except for the presence of proanthocyanidins and leucoanthocyanidins in ChL. CR showed a higher phenolic and flavonoid content and a lower hemolytic activity than ChL (*p* < 0.05). Interestingly, a negative correlation was found between their MIC values (*r* = −0.21), whereas the inhibition order was *S. aureus* > *B. subtilis* > *M. luteus* to ChL and it was *B. subtilis* > *M. luteus* > *S. aureus* to CR. In fact, all extracts from roots showed markedly inhibitory activity against *B. subtilis*. This species is a model organism present in the soil and widely known for its ability to promote plant growth, and thus has great importance in agriculture. However, little information is available about the ecological role and the mechanisms involved in the interaction with plants (Kobayashi, 2015).

On the other hand, *C. spinosa* extracts had not shown an efficacy against *Candida* spp. as good as that observed for the Gram-positive bacteria. Among all yeasts tested, *C. glabrata* and *C. tropicalis* were the most sensitive to the fungistatic action (ratio of MMC/MIC > 4). Infections caused by *Candida* spp. are especially found in immunosuppressed patients, as those with cancer, transplantation, postoperative, HIV, especially in developing countries (Pitman et al., 2011). Allied to this, the lack of new antifungals and the growing microbial resistance (Odom, 2014) also make these results relevant and important.

Therefore, due to the great potential shown by *C. spinosa* extracts against *S. aureus*, we decided to investigate the effect of cyclohexanic and chloroformic extracts against clinical isolates. The search for new anti-*S. aureus* agents is extremely important, since infections caused by multidrug resistant strains have been growing worldwide and are one of the most serious problems within health care facilities (Stryjewski and Corey, 2014). Such issues have been accounted to the excessive use of drugs (Kim et al., 2015), which results in development of new resistance mechanisms (Dordel et al., 2014). Our data shows that the chloroform extracts were able to inhibit and kill with good efficiency against different clinical strains, the most active was CL, with MIC_50_ values ranging from 4- to 8-fold lower than other extracts. It is important to note that these concentrations are much lower than their HC_50_ values. Both chloroform extracts showed bactericidal action, whereas their MIC values and MMC showed weak positive (*r* = 0.09) and moderate negative (*r* = −0.49) correlations, respectively. Then, it is possible that different mechanisms are responsible for the results observed. Furthermore, strong correlations were found between the average MIC against *S. aureus* and their phenolic (*r* = −0.89) and flavonoid content (*r* = −0.87), reinforcing the possible role of these metabolite classes on the antimicrobial activity of *C. spinosa* derived extracts.

In addition, given the importance of the *S. aureus* pathogenicity and its ability to acquire resistance, new ways to combat this pathogen must be developed, among them is combination therapy using compounds that act or not on the same target. Therefore, to test the combining action became a key step in phytochemical studies (Wagner and Ulrich-Merzenich, 2009; Bessa et al., 2015; dos Santos et al., 2015). CL and CR extracts were able to increase the effectiveness of this antibiotic, mainly through synergistic interaction. Some plant-derived products have demonstrated the ability to reverse resistance to oxacillin (Jenkins and Cooper, 2012; Bessa et al., 2015), and the perspective is that the use of therapies based on the combination of phytochemicals and antibiotics grow in conventional medicine, as it may reduce the likelihood of dose-dependent toxicity and mutagenicity of antimicrobials (Boucher and Tam, 2006; Wagner and Ulrich-Merzenich, 2009).

## Conclusion

This research demonstrates the antimicrobial potential of leaves and roots from *C. spinosa*. These chloroform and cyclohexane extracts were especially active against Gram-positive organisms, and they inhibited *S. aureus* strains with different phenotypes of resistance. Similarly, the most active extracts increased the action of oxacillin against different *S. aureus* strains. The next steps are focused on the isolation and identification of the active (s) compound (s) in each extract which could be used as lead(s) molecule (s) in the development of new antimicrobial drugs.

## Author contributions

AS, MC, VD designed the study protocol, and participated in its design and coordination. AS, JA, MS carried out the antimicrobial assays. AS, LS, CF, MS, VD contributed to drafting the manuscript and/ or critically revising the paper and intellectual content. All authors read and approved the final manuscript.

### Conflict of interest statement

The authors declare that the research was conducted in the absence of any commercial or financial relationships that could be construed as a potential conflict of interest.

## References

[B1] Adhikari R. P. Ajao A. O. Aman M. J. Karauzum H. Sarwar J. Lydecker A. D.. (2012). Lower antibody levels to *Staphylococcus aureus* exotoxins are associated with sepsis in hospitalized adults with invasive *Staphylococcus aureus* infections. J. Infect. Dis. 206, 915–923. 10.1093/infdis/jis462 22807524

[B2] Agra M. F. França P. F. Barbosa-Filho J. M. (2007). Synopsis of the plants known as medicinal and poisonous in Northeast of Brazil. Rev. Bras. Farmacogn. 17, 114–140. 10.1590/S0102-695X2007000100021

[B3] Albarello N. Simões-Gurgel C. Castro T. C. Gayer C. R. M. Coelho M. G. P. Moura R. S. (2013). Anti-inflammatory and antinociceptive activity of field-growth plants and tissue culture of *Cleome spinosa* (Jacq) in mice. J. Med. Plants. Res. 7, 1043–1049. 10.5897/JMPR12.153

[B4] Amorozo M. C. D. M. (2002). Uso e diversidade de plantas medicinais em Santo Antônio do Leverger, MT, Brasil. Acta Bot. Bras. 16, 189–203. 10.1590/S0102-33062002000200006

[B5] Andrade F. D. Ribeiro A. R. C. Medeiros M. C. Fonseca S. S. Athayde A. C. R. Ferreira A. F. (2014). Anthelmintic action of the hydroalcoholic extract of the root of *Tarenaya spinosa* (Jacq) Raf. for *Haemonchus contortus* control in sheep. Pes. Vet. Bras. 34, 942–946. 10.1590/S0100-736X2014001000003

[B6] Bessa L. J. Palmeira A. Gomes A. S. Vasconcelos V. Sousa E. Pinto M.. (2015). Synergistic Effects between thioxanthones and oxacillin against methicillin-resistant *Staphylococcus aureus*. Microb. Drug Resist. 21, 404–415. 10.1089/mdr.2014.0162 25789724

[B7] Bose A. Gupta J. K. Dash G. K. Ghosh T. SI S. Panda D. S. (2007). Diuretic and antibacterial activity of aqueous extract of *Cleome rutidosperma*. Indian J. Pharm. Sci. 69, 292–294. 10.4103/0250-474X.33162

[B8] Boucher A. N. Tam V. H. (2006). Mathematical formulation of additivity for antimicrobial agents. Diagn. Microbiol. Infect. Dis. 55, 319–325. 10.1016/j.diagmicrobio.2006.01.024 16626903

[B9] Briers Y. Lavigne R. (2015). Breaking barriers: expansion of the use of endolysins as novel antibacterials against Gram-negative bacteria. Future Microbiol. 10, 377–390. 10.2217/fmb.15.8 25812461

[B10] Costa A. F. (2002). Farmacognosia. Lisboa: Fundação Calouste Gulbenkian.

[B11] Costa D. A. Chaves M. H. Silva W. C. S. Costa C. L. S. (2010). Constituintes químicos, fenóis totais e atividade antioxidante de *Sterculiastriata* St. Hil. et Naudin. Acta Am. 40, 207–212. 10.1590/S0044-59672010000100026

[B12] Da Silva L. C. N. Sandes J. M. de Paiva M. M. de Araújo J. M. Figueiredo R. C. B. Q. D. da Silva M. V.. (2013). Anti-*Staphylococcus aureus* action of three Caatinga fruits evaluated by electron microscopy. Nat. Prod. Res. 27, 1492–1496. 10.1080/14786419.2012.722090 22974409

[B13] Dordel J. Kim C. Chung M. de la Gándara M. P. Holden M. T. Parkhill J.. (2014). Novel determinants of antibiotic resistance: identification of mutated loci in highly methicillin-resistant subpopulations of methicillin-resistant *Staphylococcus aureus*. mBio 5:e01000–e01013. 10.1128/mbio.01000-e01013 24713324 PMC3993859

[B14] dos Santos A. T. B. da Silva Araújo T. F. Da Silva L. C. N. da Silva C. B. de Oliveira A. F. M. Araújo J. M.. (2015). Organic extracts from *Indigofera suffruticosa* leaves have antimicrobial and synergic actions with Erythromycin against *Staphylococcus aureus*. Front. Microbiol. 6:13. 10.3389/fmicb.2015.00013 25699022 PMC4313721

[B15] Gyawali R. Ibrahim S. A. (2014). Natural products as antimicrobial agents. Food Control 46, 412–429. 10.1016/j.foodcont.2014.05.047

[B16] Harborne J. B. (1998). Phytochemical Methods. London: Chapman & Hall.

[B17] Harvey A. L. Edrada-Ebel R. Quinn R. J. (2015). The re-emergence of natural products for drug discovery in the genomics era. Nat. Rev. Drug Discov. 14, 111–129. 10.1038/nrd4510 25614221

[B18] Hayek S. A. Gyawali R. Ibrahim S. A. (2013). Antimicrobial natural products in Microbial Pathogens and Strategies for Combating them: Science, Technology and Education, ed Méndez-Vilas A. (Badajoz: FORMATEX), 910–921.

[B19] Hemaiswarya S. Kruthiventi A. K. Doble M. (2008). Synergism between natural products and antibiotics against infectious diseases. Phytomedicine 15, 639–652. 10.1016/j.phymed.2008.06.008 18599280

[B20] Jenkins R. E. Cooper R. (2012). Synergy between oxacillin and manuka honey sensitizes methicillin-resistant *Staphylococcus aureus* to oxacillin. J. Antimicrob. Chemother. 67, 1405–1407. 10.1093/jac/dks071 22382468

[B21] Kim J. Sudbery P. (2011). *Candida albicans*, a major human fungal pathogen. J. Microbiol. 49, 171–177. 10.1007/s12275-011-1064-7 21538235

[B22] Kim N. H. Koo H. L. Choe P. G. Cheon S. Kim M. S. Lee M. J.. (2015). Inappropriate continued empirical vancomycin use in a hospital with a high prevalence of methicillin-resistant *Staphylococcus aureus*. Antimicrob. Agents Chemother. 59, 811–817. 10.1128/AAC.04523-14 25403664 PMC4335878

[B23] Kobayashi K. (2015). Plant methyl salicylate induces defense responses in the rhizobacterium *Bacillus subtilis*. Environ. Microbiol. 17, 1365–1376. 10.1111/1462-2920.12613 25181478

[B24] Li A. B. Wonga C. C. Ka-Wing C. Chen F. (2008). Antioxidant properties in vitro and total phenolic contents in methanol extracts from medicinal plants. LWT-Food Sci. Technol. 41, 385–390. 10.1016/j.lwt.2007.03.011

[B25] McNeil M. J. Porter R. B. Williams L. A. Rainford L. (2010). Chemical composition and antimicrobial activity of the essential oils from *Cleome spinosa*. Nat. Prod. Commun. 5, 1301–1306. 20839641

[B26] Odds F. C. (2003). Synergy, antagonism, and what the chequerboard puts between them. J. Antimicrob. Chem. 52, 1–1. 10.1093/jac/dkg301 12805255

[B27] Odom A. R. (2014). The triphenylethylenes, a novel class of antifungals. mBio 5:e01126–14. 10.1128/mBio.01126-14 24781746 PMC4010834

[B28] Oliveira Y. L. C. Silva L. C. N. Silva A. G. Macedo A. J. Araújo J. M. Correia M. T. S. (2012). Antimicrobial activity and phytochemical screening of *Buchenavia tetraphylla* (Aubl) R. A. Howard (Combretaceae: Combretoideae). Sci. World J. 6:849302 10.1100/2012/849302 PMC3541636 23365533

[B29] Opperman T. J. Nguyen S. T. (2015). Recent advances toward a molecular mechanism of efflux pump inhibition. Front. Microbiol. 6:421. 10.3389/fmicb.2015.00421 25999939 PMC4419859

[B30] Pankey G. A. Sabath L. D. (2004). Clinical relevance of bacteriostatic versus bactericidal mechanisms of action in the treatment of Gram-positive bacterial infections. Clin. Infect. Dis. 38, 864–870. 10.1086/381972 14999632

[B31] Pitman S. K. Drew R. H. Perfect J. R. (2011). Addressing current medical needs in invasive fungal infection prevention and treatment with new antifungal agents, strategies and formulations. Expert. Opin. Emerg. Drugs. 16, 559–586. 10.1517/14728214.2011.607811 21846302

[B32] Robertson E. H. Cartwright R. A. Wood D. J. M. (1957). The flavones of tea. J. Sci. Food. Agr. 7, 637–646. 10.1002/jsfa.2740071003

[B33] Russell C. R. Morris D. A. (1982). Invertase activity, soluble carbohydrates and inflorescence development in the tomato (*Lycopersicon esculentum* Mill). Ann. Bot. 49, 89–98.

[B34] Simonetti G. Santamaria A. R. D'Auria F. D. Mulinacci N. Innocenti M. Cecchini F.. (2014). Evaluation of Anti-*Candida* activity of *Vitis vinifera* L. seed extracts obtained from wine and table cultivars. BioMed. Res. Int. 2014, 2314–6141. 10.1155/2014/127021 24864227 PMC4017847

[B35] Stryjewski M. E. Corey G. R. (2014). Methicillin-resistant *Staphylococcus aureus*: an evolving pathogen. Clin. Infect. Dis. 58, S10–S19. 10.1093/cid/cit613 24343827

[B36] Sudhakar M. Rao C. V. Rao P. M. Raju D. B. (2006). Evaluation of antimicrobial activity of *Cleome viscosa* and *Gmelina asiatica*. Fitoterap. 77, 47–49. 10.1016/j.fitote.2005.08.003 16325351

[B37] Taguri T. Tanaka T. Kouno I. (2006). Antibacterial spectrum of plant polyphenols and extracts depending upon hydroxyphenyl structure. Biol. Pharm. Bull. 29, 2226–2235. 10.1248/bpb.29.2226 17077519

[B38] Tegos G. Stermitz F. R. Lomovskaya O. Lewis K. (2002). Multidrug pump inhibitors uncover remarkable activity of plant antimicrobials. Antimicrob Agents Chemother. 46, 3133–3141. 10.1128/AAC.46.10.3133-3141.2002 12234835 PMC128777

[B39] Tian F. Li B. Ji B. Zhang G. Luo Y. (2009). Identification and structure–activity Relationship of gallotannins separated from *Galla chinensis*. LWT-Food Sci. Technol. 42, 1289–1295. 10.1016/j.lwt.2009.03.004

[B40] Venter H. Mowla R. Ohene-Agyei T. Ma S. (2015). RND-type drug eux pumps from Gram-negative bacteria: molecular mechanism and inhibition. Front. Microbiol. 28:377 10.3389/fmicb.2015.00377 PMC4412071 25972857

[B41] Vuuren S. Viljoen A. (2011). Plant-based antimicrobial studies – methods and approaches to study the interaction between natural products. Plant Med. 77, 1168–1182. 10.1055/s-0030-1250736 21283954

[B42] Wagner H. Bladt S. (1996). Plant Drug Analysis: A Thin Layer Chromatography Atlas. Berlin: Springer Science Business Media.

[B43] Wagner H. Ulrich-Merzenich G. (2009). Synergy research: approaching a new generation of phytopharmaceuticals. Phytomedicine 16, 97–110. 10.1016/j.phymed.2008.12.018 19211237

[B44] Woisky R. G. Salatino A. (1998). Analysis of propolis: some parameters and procedures for chemical quality control. J. Apic. Res. 37, 99–105.

[B45] World Health Organization (2014). Antimicrobial Resistance: Global Report on Surveillance. Geneva: World Health Organization.

